# COVID-19 should be a methylene blue “promoter”

**DOI:** 10.21470/1678-9741-1-2020-0607

**Published:** 2020

**Authors:** Paulo Roberto B. Evora

**Affiliations:** 1 Department of Surgery and Anatomy Ribeirão Preto School of Medicine. Faculdade de Medicina de Ribeirão Preto da Universidade de São Paulo (FMRP-USP), Ribeirão Preto, SP, Brazil. E-mail:prbevora@gmail.com

**To the Editor,**

Since the seventies, when I started my intensive care and cardiac surgery duties, I continue with total non-conformity in the face of death, especially when significant battles are lost, for example, against septic shock in young women with gynecological infections, as well as patients who die from anaphylactic shock. Even with the constant and growing commitment to saving lives, like those of the young people mentioned above, we continue to lose the battle for infection/inflammation.

Right now, we are fighting against the COVID-19 pandemic, whose physiopathology surely includes inflammation and the NO-cGMP endothelium-dependent vasoplegic dysfunction. Some well-known observations should be mentioned over and over, based on what has been learned about blocking the NO-cGMP pathway in the treatment of vasoplegic endothelial dysfunction for synthesizing concepts:

1. The use of methylene blue (MB) does not cause endothelial dysfunction.
2. The MB effect appears in cases without positive NO regulation.
3. MB itself is not a vasoconstrictor. By blocking the cGMP pathway, it releases the cAMP pathway, facilitating the vasoconstrictor effect of epinephrine through this “crosstalk” mechanism.
4. The most used dosage is 2 mg/kg in IV bolus, followed by the same continuous hourly infusion. The plasma concentration declines sharply in the first 40 minutes.
5. MB has an antioxidant effect.

Based on these concepts, we keep saving lives, and with the certainty that the NO-cGMP pathway blocking by MB has still underestimated at least for more than 100 years. However, many health professionals named MB as a “rescue magic bullet.”

I saw COVID-19 inside this scenario and, last March, I wrote a letter to the *Lancet’s* Editor-in-Chief entitled “SHOULD METHYLENE BLUE CONSIDERED AS ‘RESCUE MAGIC BULLET’ AGAINST THE NEW CORONAVIRUS?”

The MB/light-based method has been used routinely in Europe for about 17 to 18 years. Plasma units from blood donations are illuminated with visible light in the presence of MB. The MB/light-treated generates singlet oxygen, which leads to the destruction of viral nucleic acids. Emerging groups include severe acute respiratory syndrome coronavirus (SARS-CoV), Crimean-Congo hemorrhagic fever virus (CCHFV) and Nipah virus (NiV), which have been identified by the World Health Organization (WHO) as major infectious threats with the potential to cause a global pandemic^[1-3]^. Paul Ehrlich, obsessed with structural organic chemistry and dyes, elaborated his theory regarding the discovery of a “magic bullet”. Based on all of his scientific discoveries, he won the Nobel Prize in 1908, with an emphasis on the treatment of malaria with MB. Should MB, a precursor to hydroxychloroquine, be a “rescue magic bullet” against the new coronavirus? If someone chooses to test the idea, I suggest, as a therapeutic test, an initial IV bolus of 1 mg/ kg. In our experience in the treatment of vasoplegic syndrome, the highest dose is 7 mg/kg in continuous IV infusion^[4]^.

**Fig. 1:**
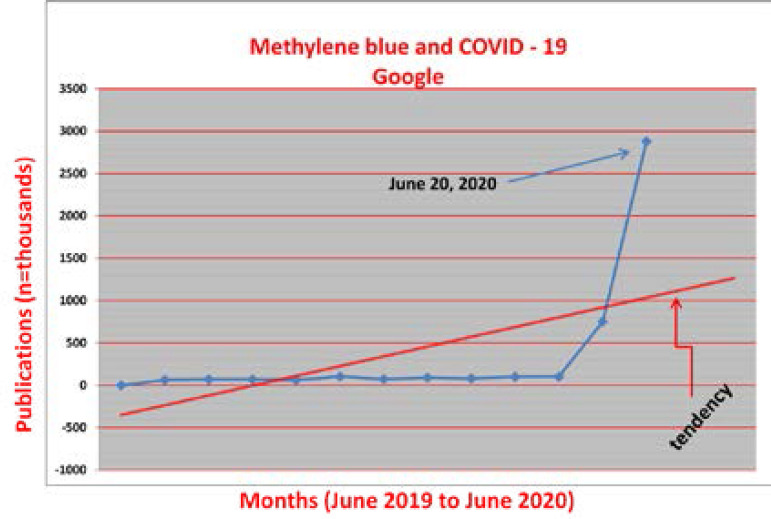
Evolution of the overall number of citations compiled as a result of a generic consultation (methylene blue and COVID-19) (Google).

The Lancet Global Health, on this occasion, decided not to publish the letter because they believe “the message would be better suited elsewhere”. I agreed with the decision and, during my COVID-19 quarantine, I kept my routine Google consulting “methylene blue and COVID-19”. Suddenly… an explosion of almost three million papers (Figure 1).

At that time, two philosophical quotes motivated me to rewrite the rejected letter: “Medicine is the science of uncertainty and the art of probability” (William Osler) and “Nothing belongs to you more than your dreams” (Friedrich Nietzsche). I decided to resubmit the letter now, considering that COVID-19 should be a methylene blue “promoter”, and the dye can get the lifesaving status it deserves.
